# Glycycoumarin Sensitizes Liver Cancer Cells to ABT-737 by Targeting De Novo Lipogenesis and TOPK-Survivin Axis

**DOI:** 10.3390/nu10030353

**Published:** 2018-03-15

**Authors:** Enxiang Zhang, Shutao Yin, Xiaotong Lu, Linhu Ye, Lihong Fan, Hongbo Hu

**Affiliations:** 1 Beijing Advanced Innovation Center for Food Nutrition and Human Health, College of Food Science and Nutritional Engineering, China Agricultural University, No. 17 Qinghua East Road, Haidian District, Beijing 100083, China; zhangenxiang0728@gmail.com (E.Z.); yinshutao@cau.edu.cn (S.Y.); luxiaotong@cau.edu.cn (X.L.); yelinhu@126.com (L.Y.); 2 College of Veterinary Medicine, China Agricultural University, No. 2 Yuanmingyuan West Road, Haidian District, Beijing 100193, China; 04055@cau.edu.cn

**Keywords:** BH3 mimetics, ABT-737, liver cancer, sensitization, Glycycoumarin, TOPK, survivin, de novo lipogenesis

## Abstract

Glycycoumarin (GCM) is a representative of bioactive coumarin compounds isolated from licorice, an edible and medicinal plant widely used for treating various diseases including liver diseases. The purpose of the present study is to examine the possibility of GCM as a sensitizer to improve the efficacy of BH3 mimetic ABT-737 against liver cancer. Three liver cancer cell lines (HepG2, Huh-7 and SMMC-7721) were used to evaluate the in vitro combinatory effect of ABT-737/GCM. HepG2 xenograft model was employed to assess the in vivo efficacy of ABT-737/GCM combination. Results showed that GCM was able to significantly sensitize liver cancer cells to ABT-737 in both in vitro and in vivo models. The enhanced efficacy by the combination of ABT-737 and GCM was attributed to the inactivation of T-LAK cell-originated protein kinase (TOPK)-survivin axis and inhibition of de novo lipogenesis. Our findings have identified induction of TOPK-survivin axis as a novel mechanism rendering cancer cells resistant to ABT-737. In addition, ABT-737-induced platelet toxicity was attenuated by the combination. The findings of the present study implicate that bioactive coumarin compound GCM holds great potential to be used as a novel chemo-enhancer to improve the efficacy of BH3 mimetic-based therapy.

## 1. Introduction

Disruption of mitochondrial membrane potential (MMP) is one of the key events in the apoptotic process [1]. MMP is tightly regulated by Bcl-2 family proteins including anti-apoptotic members of this family Bcl-2, Bcl-xl, Mcl-1, and pro-apoptotic members of this family Bax, Bak, Bim, Puma, Bid and Bad etc. [2]. The evasion of apoptosis is one of the hallmarks of cancer cells, which is commonly associated with aberrant expression of anti-apoptotic Bcl-2 family members [3]. Apoptosis defects not only promote cancer cell survival, but also render cancer cells refractory to therapeutic drugs [4,5]. Therefore, these Bcl-2 family anti-apoptotic proteins are considered to be rational targets for targeted cancer treatments. To this end, several small molecule inhibitors of anti-apoptotic Bcl-2 family proteins named BH3 mimetics have been developed [6]. These inhibitors can specifically bind to the hydrophobic groove of the anti-apoptotic Bcl-2 family proteins, leading to inhibition of their anti-apoptotic function and followed by activation of mitochondrial pathway. Among these inhibitors, ABT-737 and its orally available derivative ABT-263 are relatively well investigated [7,8]. Previous studies have demonstrated that ABT-737 is a promising anti-cancer agent against diverse types of cancer including myeloma, leukemia, glioblastoma, liver, lung, and prostate cancer [9,10,11,12,13,14]. The major obstacles for the therapeutic use of ABT-737 and its analog ABT-263 are Mcl-1 induction and Bcl-2 phosphorylation-mediated resistance and dose-limiting toxicity on platelet [15]. To deal with these issues, combining ABT-737 with the agents that can either avert the problem of resistance or reduce the adverse effect is suggested to be a practical approach. 

Licorice has been used in Chinese herbal medicine for centuries to treat various diseases. Glycycoumarin (GCM) is a major coumarin compound isolated from licorice [16]. Previous studies by us or others have demonstrated that GCM possess multiple biological activities including anti-cancer effects [17]. In addition, the pharmacokinetic study by Ye’s group has shown that GCM has favorable oral bioavailability [18]. In the present study, we aimed to address the possibility of GCM as a sensitizer to improve efficacy of ABT-737 against liver cancer using both in vitro and in vivo models. Results showed that GCM was able to significantly increase the efficacy of ABT-737 against liver cancer in both cell culture and xenograft animal models with reduced platelet toxicity. Mechanistically, we revealed that the enhanced efficacy by the combination of ABT-737 and GCM was attributed to the inactivation of TOPK-survivin axis and the inhibition of de novo lipogenesis. Our findings support GCM as an effective enhancer to improve the efficacy of ABT-737. 

## 2. Materials and Methods

### 2.1. Chemicals and Reagents

GCM (purity > 98%) was isolated from the rhizomes of glycyrrhiza using a previously reported method [16]. ABT-737 was provided by Abbott Laboratories (Abbott Park, IL, USA). Orlistat was purchased from Med Chem Express (Danvers, MA, USA). The primary antibodies of TOPK, p-TOPK, p-AMPK (AMP-activated protein kinase), p-ACC (Acetyl-CoA carboxylase), survivin, p-survivin, Mcl-1 and p-H3 were purchased from Cell Signaling Technology (Danvers, MA, USA). Primary antibody specific for β-actin was purchased from Action Biotech (Beijing, China). The second-antibody specific for Rabbit and Mouse were purchased from MBL International Corporation (Boston, MA, USA). *PBK/TOPK-siRNA* and *control-siRNA* were purchased from life technology (Waltham, MA, USA). *Survivin-siRNA* was purchased from Santa Cruz Biotechnology (Santa Cruz, CA, USA). YM155 was purchased from Med Chem Express.

### 2.2. Apoptosis Evaluation

Apoptosis was determined by flow cytometry following Annexin V/PI double staining of externalized phosphatidyl-serine (PS) in apoptotic cells using Annexin V/PI staining kit from MBL International Corporation (Boston, MA, USA).

### 2.3. Calculation of Combination Index

The synergistic effects between ABT-737 and GCM were quantitatively assessed by calculation of combination index (CI) using Chou–Talalay equation [19]. The cells were treated with various concentrations of ABT-737, GCM and their combination. The overall inhibitory effect was evaluated by Crystal Violet Staining described above. Combination index (CI) was calculated using the following Equation (1):

(1) $$CI\;=\;\frac{CA,\;x}{ICx,\;A}\;+\;\frac{CB,\;x}{ICx,\;B}$$

where *CA*, x and *CB*, x are the concentrations of agent *A* and agent *B* used in combination to achieve x % combinatory effect; *IC*x, *A* and *IC*x, *B* are the concentrations for single agent to achieve the same effect. CI < 1, CI = 1 and CI > 1 indicate synergism, additive effect and antagonism respectively.

### 2.4. Western Blotting

The cell was lysed with ice-cold RIPA (radio-immuno-precipitation assay) buffer with protease inhibitor. Equal amount of proteins of the samples was loaded onto the gel. After electrophoretic separation, the proteins were transferred to a nitrocellulose membrane. The membrane was subsequently probed with primary antibodies following the incubation with corresponsive secondary antibody. The immune-reactive blots were visualized using enhanced chemi-luminescence. 

### 2.5. RNA Interference

The cells were transfected with 7.5 nM of *PBK/TOPK-siRNA* and 50 nM *survivin-siRNA* or *negative control siRNA* using INTERFERin siRNA transfection reagent according to the manufacturer’s instructions (Polyplus-Transfection, Illkirch, France). 24 h post-transfection, the cells were used for subsequent experiments.

### 2.6. Animal Study

The in vivo combinatory anti-cancer activity of GCM and ABT-737 were evaluated using HepG2 xenograft model. Animal Care and experimental protocols were approved by the Institutional Animal Care and Use Committee (China Agricultural University). Mice were housed in a pathogen-free barrier facility accredited by the Association for Assessment and all animal procedures were carried out in accordance with institutional guidelines for animal research. To establish the cancer xenograft, 2 × 10^6^ HepG2 cells were mixed with Matrigel (50%) (Becton Dickinson, NJ, USA) and injected subcutaneous (s.c.) into the right flank of 6–7-week-old male BALB/c athymic nude mice (Charles River Laboratories). Tumors were measured with a caliper and tumor volumes were calculated using the following formula: 1/2(*w*1 × *w*2 × *w*2), where *w*1 is the largest tumor diameter and *w*2 is the smallest tumor diameter. When the tumor volume was up to about 100–120 mm^3^, GCM (10 mg/kg), Orlistat (100,150 mk/kg) and ABT-737(100 mg/kg) were given by intraperitoneal injection. GCM was given every day, ABT-737 and Orlistat were given every two days. GCM and Orlistat were dissolved with 5% tween 80. ABT-737 was dissolved with 30% propylene glycol, 5% Tween 80 and 65% D5W (5% dextrose in water). The body weight and tumor volume were evaluated every other day. The tumor tissues were collected and stored in −80 °C.

### 2.7. Measurement of Platelets Concentration

Mice were treated with 10 mg/kg GCM for 4 days and then 100 mg/kg ABT-7373 was given. Blood was collected after 4 h ABT-737 treatment and platelet concentration was assessed via blood routine test. This experimental design was based on the ref [15], in which a single IP injection of ABT-737 resulted in a significant reduction in platelet count within 4 h of administration. 

### 2.8. Statistical Analysis

Data were presented as mean ± SD (standard derivation). These data were analyzed with the ANOVA with appropriate post-hoc comparison among means with Graph Pad Prism 6.0 (GraphPad Software, San Diego, CA, USA). 

## 3. Results

### 3.1. Combining GCM with ABT-737 Synergistically Induces Cell Death in Multiple Types of Liver Cancer Cells

**Figure 1:**
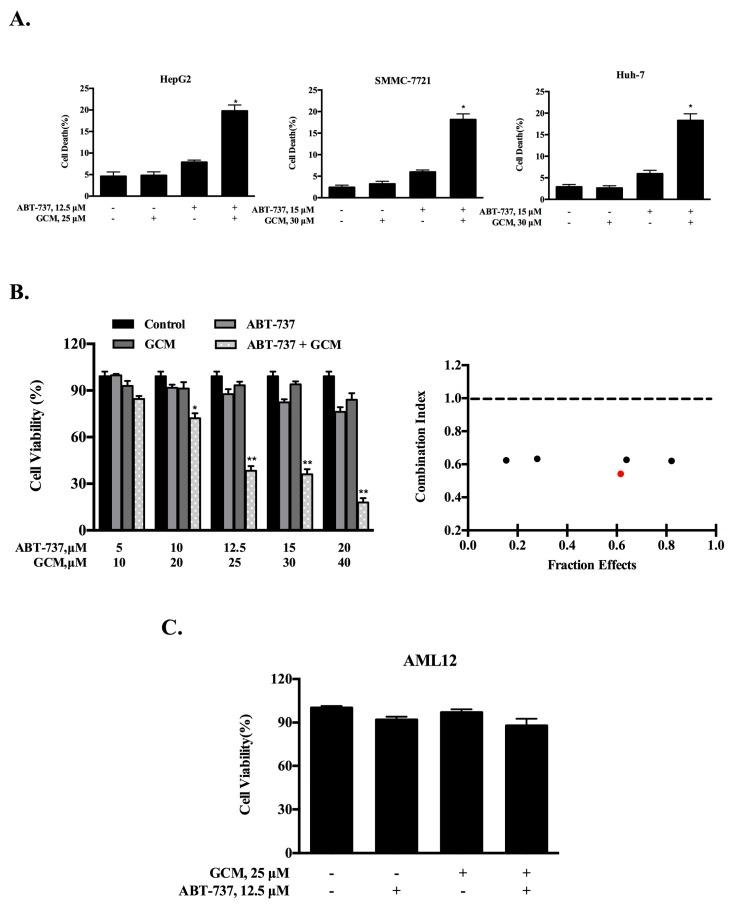
Combining GCM with ABT-737 synergistically induces cell death in multiple types of liver cancer cells. (**A**) HepG2, SMMC-7721 and Huh-7 cells were treated with GCM, ABT-737 or their combination for the indicated times and the cell death was measured via flow cytometry; (**B**) HepG2 cells were treated with ABT-737, GCM, or the combination in a fixed ratio (1:2) for 24 h and the cell viability was assessed by crystal violet staining; the combination index of the combination was calculated using Chou–Talalay equation based on the data generated above (red dot represents that this combination was used in the subsequent experiments); (**C**) Inhibitory effects of GCM and/or ABT-737 on AML12 cells measured by crystal violet staining. GCM: Glycycoumarin. *p* < 0.05 (*), *p* < 0.005 (**).

To evaluate the combination effect of GCM and ABT-737 on liver cancer cells, HepG2 cells were first employed. The cells were treated with each agent alone or their combination at the concentrations indicated for 24 h and cell death was measured by Annexin v/PI staining. As shown in Figure 1A (left), exposure to 25 µM GCM did not increase cell death induction, whereas treatment with 12.5 µM ABT-737 caused a modest increase of cell death. Treatment with their combination resulted in a significantly enhanced cell death induction in HepG2 cells. To confirm this enhancement, two additional liver cancer cell lines SMMC-7721 and Huh-7 were tested and similar enhanced cytotoxicity by the combination was observed in both cell lines tested (Figure 1A, middle and right). We next asked whether the enhanced effect was a synergistic action. HepG2 cells were treated with each agent alone or their combination in a fixed ratio of 2:1 (GCM:ABT-737) for 24 h and the cell viability was detected by crystal violet staining. As shown in Figure 1B (left), treatments with GCM (10–40 µM) or ABT-737 (5–20 µM) individually caused a dose-dependent inhibitory influence on cell viability, whereas the combinations induced significantly further decreased cell viability. The data were then used to calculate the combination index based on the Chou-Talalay method [19] and the result are shown in Figure 1B (right). The values of the combination index were less than 1 at all combinations tested, supporting a synergistic nature of the combination effect on liver cancer cells by GCM/ABT-737. To examine if the potentiated effect can also be found in non-malignant hepatocytes, we measured the changes of cell viability in response to GCM and/or ABT-737 in AML12 cells. As shown in Figure 1C, the enhanced effect was not detected by the same treatments in these non-tumorigenic mouse liver cells. These data suggest that the sensitization effect maybe specific for the tumor cells. 

### 3.2. Co-Treatment of GCM and ABT-737 Results in Enhanced Tumor Growth Inhibition in Hepg2 Xenograft Model

**Figure 2:**
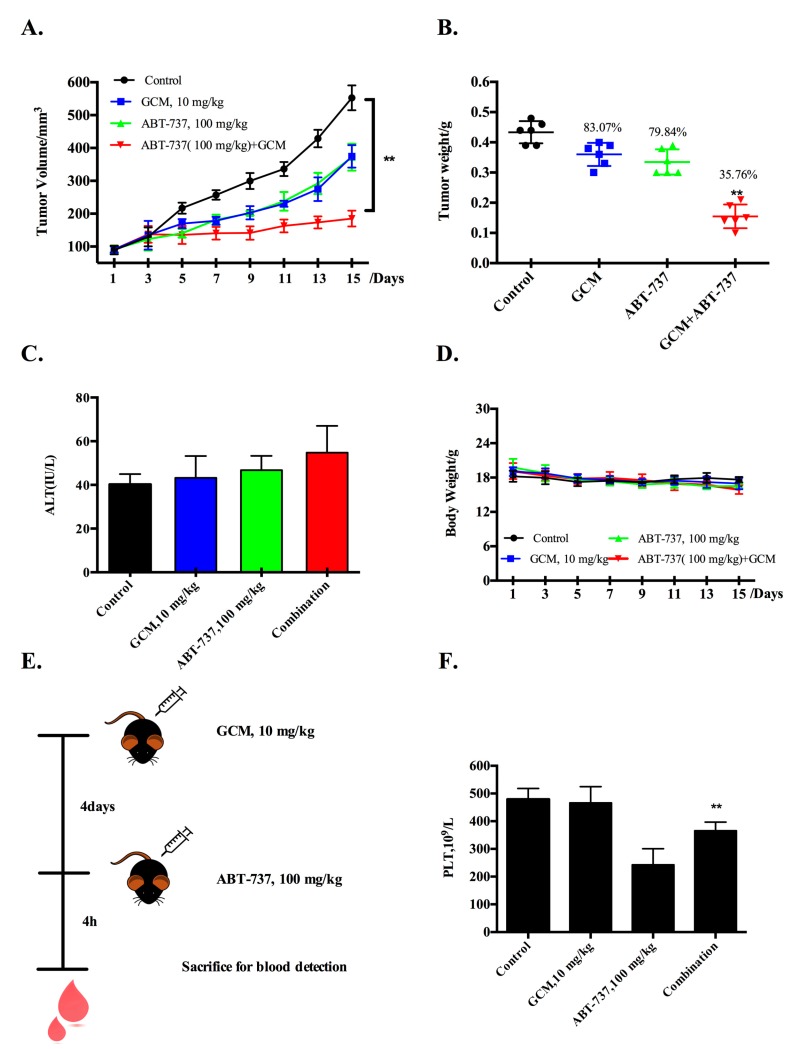
Co-treatment of GCM and ABT-737 results in enhanced tumor growth inhibition in HepG2 xenograft model. (**A**) Inhibitory effects of GCM, ABT-737 and the combination on tumor growth; (**B**) Reduction of the final tumor weight; (**C**) Serum levels of ALT; (**D**) Body weight kinetics of mice; (**E**,**F**) Influences of ABT-737, GCM and the combination on platelet count. *p* < 0.05 (*), *p* < 0.005 (**).

Having found the synergistic effect of GCM/ABT-737 combination in the cell culture model, we next questioned if the enhancement action can be achieved in vivo. Treatments were initiated when the average tumor volume reached about 100 mm^3^ as described in the Materials and methods. To increase the likelihood of detecting an enhanced combinatory effect, we used the doses of each agent alone that by themselves caused a modest tumor reduction based on our dose-finding experiment. As shown in Figure 2A,B, treatments with ABT-737 (100 mg/kg body weight, every two days) significantly inhibit tumor growth, leading to reduction of the final tumor weight by 20.1%, whereas a comparable inhibitory effect was achieved by the daily treatment with GCM (10 mg/kg body weight). Combining ABT-737 with GCM resulted in a further enhanced inhibitory effect on tumor growth, leading to decrease of the final tumor weight by 64.2%. The serum levels of ALT, a key biochemical marker of hepatotoxicity, were not significantly increased in the combination-treated mice compared with that found in ABT-737 treatment alone (Figure 2C). Bodyweight did not show difference among the treatment groups (Figure 2D). These results suggest that the combination was well tolerated by the mice. The data indicated that improvement of the therapeutic efficacy of ABT-737 in vivo can be accomplished by combining it with GCM.

Having found the improved efficacy of the combination, we then questioned whether the ABT-737-mediated platelet toxicity was also increased by the combination. Blood samples were collected, and platelet concentration was measured by blood routine test (Figure 2E). As shown in Figure 2F, a significant reduced platelet count was observed in ABT-737-treated blood samples, whereas this change was ameliorated significantly by combining ABT-737 with GCM, suggesting that GCM protected against ABT-737-mediated toxicity on platelets instead of increasing the toxicity.

### 3.3. Down-Regulation of Survivin Is Involved in the Cell Death Induced by the Combination of GCM and ABT-737

**Figure 3:**
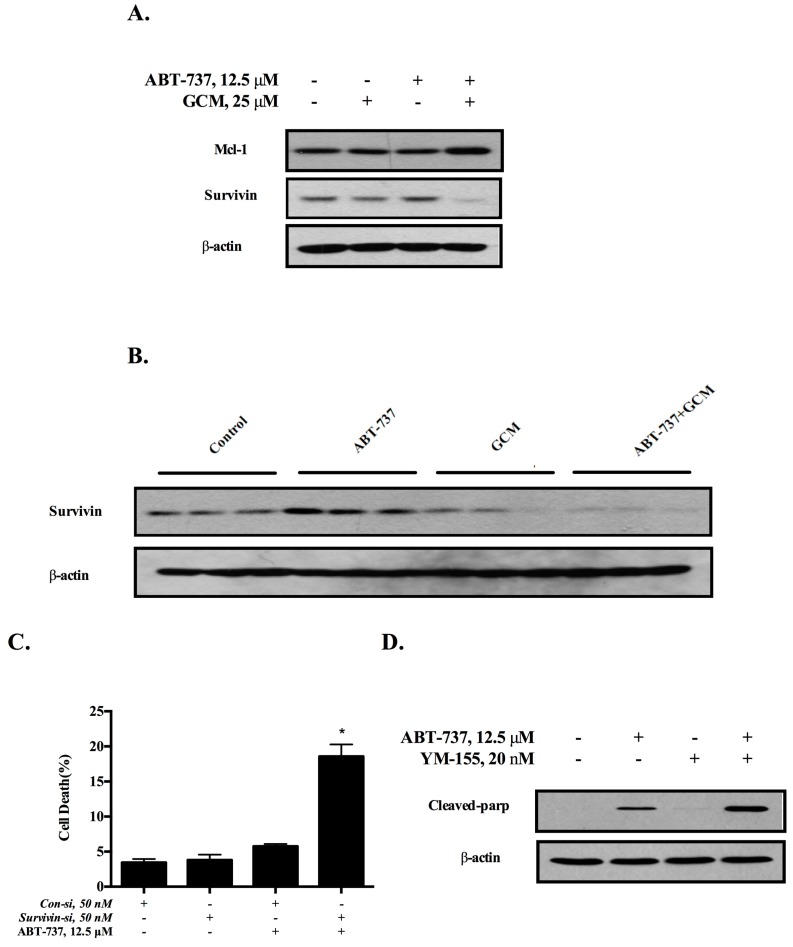
Down-regulation of survivin is involved in the cell death induced by combination of GCM and ABT-737 in HepG2 cells. (**A**) Western blot was applied to detect the expression of Mcl-1 and survivin in vitro; (**B**) Survivin expression detection in vivo via western blot; (**C**) Effects of survivin inhibition by RNAi on cell death induction by ABT-737 measured by Annexin V/PI staining; (**D**) Influences of YM155, a chemical inhibitor of survivin on ABT-737-induced apoptosis analyzed by western blot. *p* < 0.05 (*), *p* < 0.005 (**).

**Figure 4:**
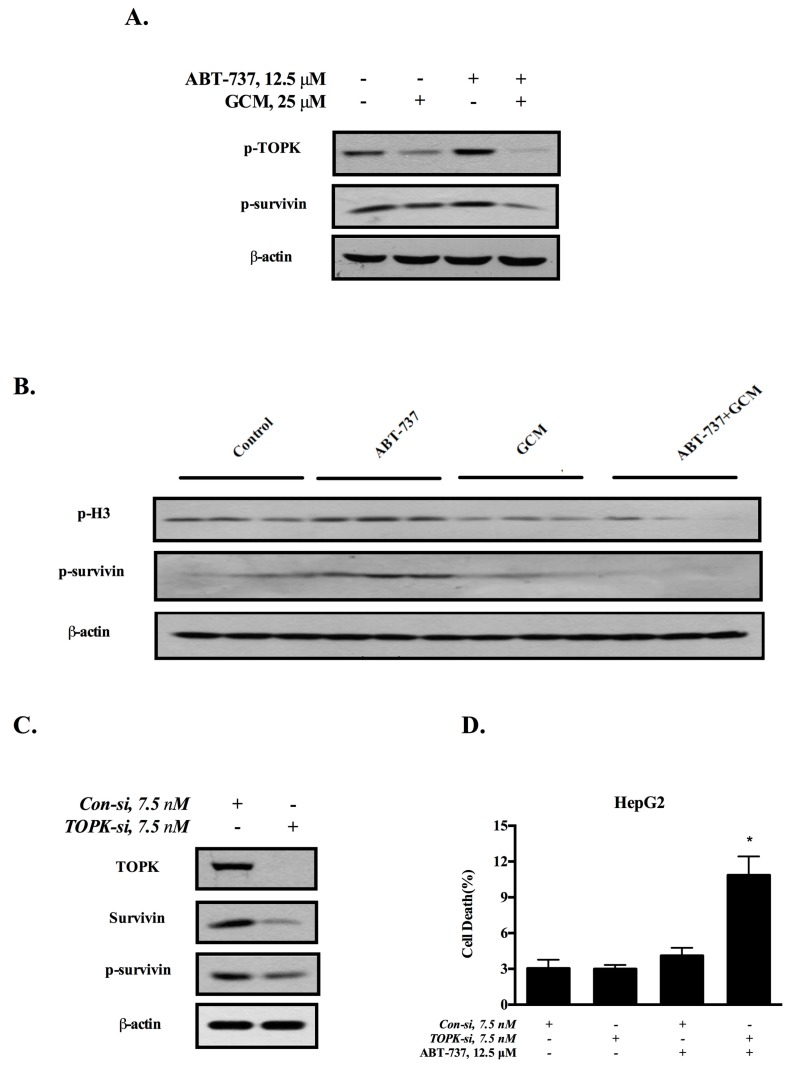
Down-regulation of survivin is attributed to the inactivation of TOPK. (**A**) Influences of GCM, ABT-737 and the combination on phosphorylation of TOPK and survivin analyzed by western blot in vitro; (**B**) Analysis of p-H3 and p-survivin in tumor samples by western blot; (**C**) Effects of TOPK knockdown on survivin expression; (**D**) Influences of TOPK knockdown on cell death induction by ABT-737. *p* < 0.05 (*), *p* < 0.005 (**).

Anti-apoptotic Bcl-2 family protein Mcl-1 has been identified as an important target for sensitizing cancer cells to ABT-737 [20]. We next asked whether this is the case for the present study. As shown in Figure 3A, neither GCM alone nor the combination decreased the expression of Mcl-1 in cell culture model. However, down-regulation of survivin, a key inhibitor of apoptosis protein (IAP), was observed in response to either GCM alone or the combination, whereas ABT-737 alone caused an increased survivin expression. We further validated the changes of survivin expression in the in vivo model. As shown in Figure 3B, ABT-737 alone induced a significant up-regulation of survivin expression in tumor tissues, whereas, the expression of survivin in tumor was significantly reduced by either GCM alone or the combination. To determine the role of the down-regulation of survivin in the sensitization of cancer cells to ABT-737, we assessed the influences of survivin knockdown on the cell viability and the results are shown in Figure 4C. Knockdown of survivin resulted in a significantly increased cytotoxicity in response to ABT-737, suggesting the inhibition of survivin was sufficient to potentiate the cancer cells to ABT-737. To further confirm the role of survivin in the sensitization, we tested effect of YM155 [21], a chemical inhibitor of survivin, on ABT-737-induced apoptosis in HepG2 cells and the results demonstrated that the inhibitor significantly sensitized the cancer cells to ABT-737-induced apoptosis (cleaved PARP) (Figure 3D), Together, these results clearly indicated that down-regulation of survivin contributed to the sensitization effect of GCM.

### 3.4. Down-Regulation of Survivin Is Attributed to the Inactivation of TOPK

Our previous study has shown that GCM can directly bind to oncogenic kinase T-LAK cell-originated protein kinase (TOPK) and inhibit its kinase activity [17]. We questioned whether the down-regulation of survivin by GCM was due to its ability to inactivate TOPK. As shown in Figure 4A, ABT-737 increased level of TOPK phosphorylation, which was decreased by GCM in HepG2 cells. The changes of TOPK phosphorylation was well correlated with phosphorylation level of survivin. To validate these in vitro findings, we analyzed the changes of TOPK and survivin in the tumor samples. As shown in Figure 4B, treatment with ABT-737 led to an increased phosphorylation level of H3, a known substrate of TOPK, and survivin, whereas exposure to GCM abolished the increased phosphorylation of H3 and survivin. To determine the relationship between TOPK and survivin, we examined effect of TOPK inhibition by RNAi on the expression of survivin. As shown in Figure 4C, knockdown of TOPK resulted in decreased total and phosphorylated survivin, suggesting a role of TOPK in the regulation of survivin. Moreover, under condition of the inactivation of TOPK, HepG2 cells were more sensitive to ABT-737-induced apoptosis (Figure 4D). Together, these results suggested that the sensitization effect of GCM was attributed to its ability to suppress ABT-737-induced TOPK-survivin pro-survival signaling.

### 3.5. Inhibition of De Novo Lipogenesis Contributes to the Sensitization Effect of GCM In Vitro and In Vivo

**Figure 5:**
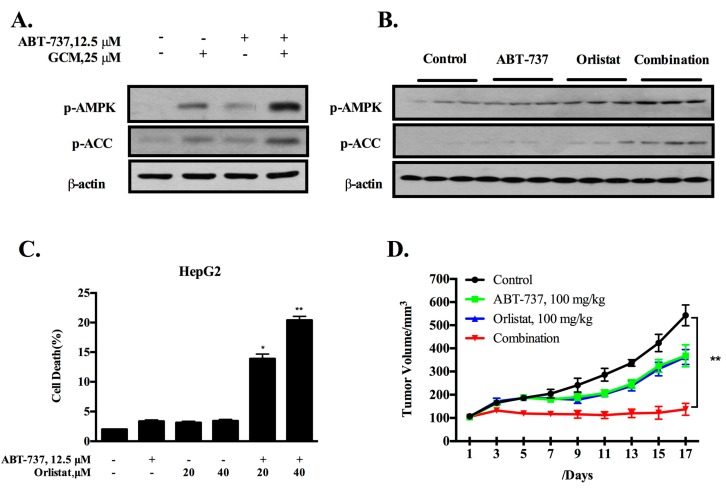

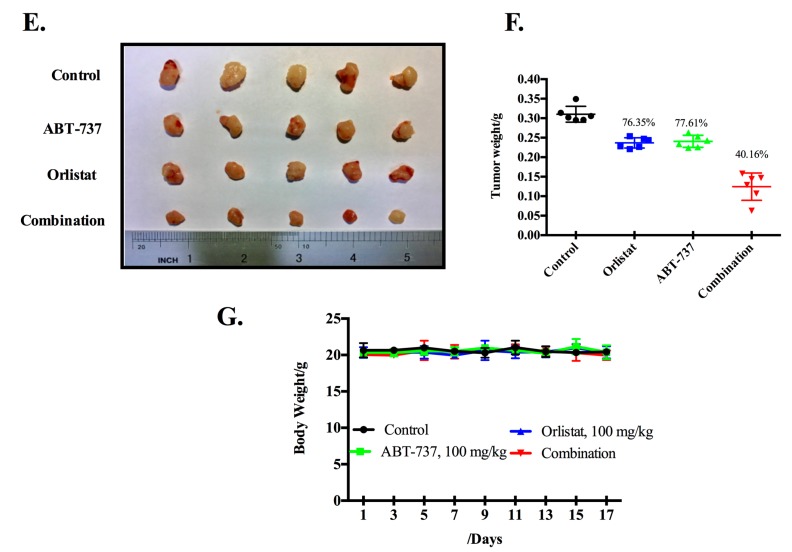
Inhibition of de novo lipogenesis contributes to sensitization effect of GCM in vitro and in vivo. (**A**,**B**) Effects of GCM, ABT-373 and the combination on phosphorylation of AMPK and its substrate ACC measured by western blot in vitro (**A**) and in vivo (**B**); (**C**) Influences of lipogenesis inhibition by orlistat on apoptosis induced by ABT-737 in HepG2 cells; (**D**) Inhibitory effect of orlistat, ABT-737 and the combination on tumor growth; (**E**,**F**) Reduction of the final tumor weight by of orlistat or/and ABT-737; (**G**) Body weight kinetics of mice. *p* < 0.05 (*), *p* < 0.005 (**).

Enhanced de novo fatty acid synthesis is a metabolic hallmark of cancer [22]. Fatty acid synthesis inhibition by targeting key lipogenic enzymes has been recognized as a promising cancer therapeutic approach [23]. Our previous study has shown that GCM can inhibit lipid accumulation in a model of non-alcoholic fatty liver disease [24]. We then asked whether the inhibition of lipogenesis by GCM contributed to its sensitization effect. HepG2 cells were treated with ABT-737 and/or GCM for the indicated times and changes of the key regulators of lipogenesis AMPK and its substrate acetyl-CoA carboxylase (ACC) were assessed to determine if GCM can inhibit fatty acid synthesis in the present setting. As shown in Figure 5A, the phosphorylation levels of AMPK and ACC (inhibitory phosphorylation) were increased by either GCM alone or the combination. Moreover, these changes were also found in the tumor samples (Figure 5B), suggesting the key lipogenic enzyme activity of ACC was inhibited in the presence of GCM. To determine the contribution of ACC inhibition to the sensitization effect, we tested the influences of orlistat, a known ACC inhibitor, on cell death induction by ABT-737 in cell culture model. As shown in Figure 5C, the cell death induction by ABT-737 was significantly increased in the presence of orlistat. We further confirmed these in vitro results in HepG2 xenograft model. The dose of orlistat (100 mg/kg) we used for the in vivo study was determined based on the dose-finding experiment. As shown in Figure 5D–F, either ABT-737 or orlistat alone significantly inhibited tumor growth, leading to reduction of the final tumor weight by approximately 20%. As expected, the combination caused a further enhanced tumor growth inhibition (Figure 5D), resulted in decrease of final tumor weight by 59.8% (Figure 5E,F) without affecting bodyweight (Figure 5G). These results suggested that the inhibition of ACC by GCM may contribute to its sensitization effect on ABT-737.

### 3.6. TOPK-Survivin Axis and AMPK-ACC Axis Are Involved Separately in the Sensitization Effect of GCM on ABT-737

**Figure 6:**
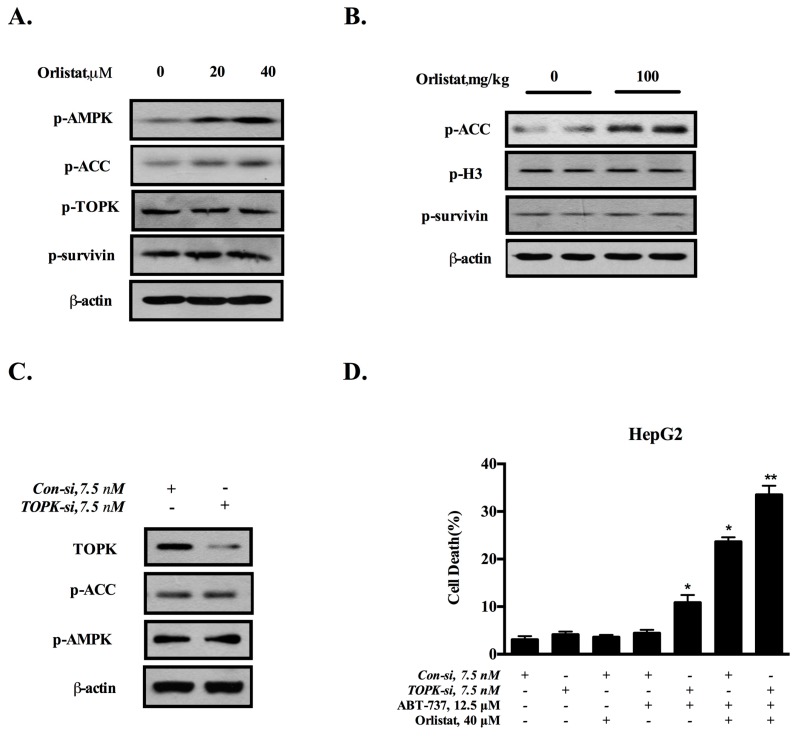
TOPK-survivin axis and AMPK-ACC axis are involved separately in sensitization effect of GCM on ABT-737. (**A**,**B**) Effects of ACC inhibitor (Oristat) on p-AMPK, p-ACC, assessed by western blot in vitro (**A**) and in vivo (**B**); (**C**) Effects of TOPK knockdown on p-AMPK and p-ACC analyzed by western blot; (**D**) Influences of TOPK knockdown and/or orlistat on apoptosis induced by ABT-737 in HepG2 cells. *p* < 0.05 (*), *p* < 0.005 (**).

The above data indicated that both TOPK-survivin and AMPK-ACC axis contributed to the sensitization effect of GCM. We next asked whether a crosstalk exists between these two pathways. The cells were treated with orlistat (20, 40 µM) for the indicated time and the changes of phosphorylation levels of ACC, TOPK and survivin were analyzed by western blotting. As shown in Figure 6A, treatment with orlistat led to increased phospho-ACC without affecting the phosphorylation status of TOPK/survivin. These in vitro results were consistent with that found in tumor tissues (Figure 6B). The data suggested that TOPK/survivin axis was unlikely the downstream target of ACC. Conversely, TOPK was silenced by RNAi and the changes of AMPK-ACC axis were examined. As shown in Figure 6C, inhibition of TOPK did not affect the phosphorylation of AMPK and ACC. Together, these data indicated that these two axes were separate to contribute to the sensitization effect of GCM. Accordingly, simultaneous inhibition of these two axes resulted in a stronger enhanced effect compared to the suppression of each axis alone (Figure 6D).

## 4. Discussion

BH3 mimetics ABT-737 and ABT-263 are representative of molecular targeted therapeutic agents with promising therapeutic efficacy. However, the drug resistance and dose-limiting toxicity pose major challenges for their clinical use [15]. The findings of the present study demonstrated that combining ABT-737 with GCM not only improved the efficacy, but also ameliorated the toxicity. Our findings therefore provided a possible new option for promoting utilization of BH3 mimetics.

Mcl-1-mediated apoptotic resistance is suggested to be the key cause of cancer cells refractory to ABT-737 [20]. Indeed, several studies have shown that targeting Mcl-1 by either chemical agents or genetic approaches resulted in a significant enhanced anti-cancer activity of ABT-737 in both in vitro and in vivo models [9,25,26]. To decipher the mechanisms involved in sensitization effect of GCM on ABT-737, we measured the influences of GCM on Mcl-1 expression and inhibitory effect of GCM on Mcl-1 was not found in the present study, ruling out the possibility of Mcl-1 as a target for GCM to exert its potentiation effect. Interestingly, we found that survivin, a key inhibitor of apoptosis protein (IAP) [27], was induced in response to ABT-737 in both in vitro and in vivo models, and this induction was abolished by combining ABT-737 with GCM. We further revealed that ABT-737-induced survivin was attributed to its ability to activate oncogenic kinase TOPK, whereas inhibition of survivin by GCM was due to its directly inactivating TOPK [17]. Moreover, the functional role of activation of TOPK-survivin axis in the regulation of apoptotic effect of ABT-737 was critically determined by the experiments that silencing TOPK or survivin was sufficient to sensitize the cancer cells to ABT-737. Our present study therefore identified induction of TOPK-survivin as a novel mechanism that contributed to cancer cells resistant to ABT-737 and targeting this axis may represent a novel approach to augment therapeutic efficacy of ABT-737. In addition, we provided the evidence that survivin was a novel downstream target of TOPK that may contribute to the tumorigenic activity of TOPK. Whether survivin is a direct substrate of TOPK is being investigated. 

Tumor growth requires continuous biosynthesis including de novo fatty acid synthesis. Enhanced de novo lipogenesis is the third metabolic feature of cancer in addition to alterations in glucose and glutamine metabolisms [22]. Among several lipogenic genes, ACC is one of the rate-limiting enzymes that control the lipogenic process [28], and elevated expression of ACC has been found in a variety of cancer types including liver cancer [22,29]. Therefore, ACC has been recognized to be a novel target for cancer treatment. Indeed, several recent studies have demonstrated that inhibition of ACC-mediated lipogenesis by either genetic or pharmacological approach offered a promising anti-cancer efficacy against a wide range of cancer in pre-clinical models [22,29,30]. Moreover, suppression of ACC can increase the sensitivity of cancer cells to certain therapeutic drugs [22,29]. However, whether inhibition of ACC-mediated lipogenesis can improve the efficacy of BH3 mimetics such as ABT-737 has not been addressed. In the present study, we investigated anti-liver cancer effect of combining ABT-737 with orlistat using both in vitro and in vivo models and results demonstrated that a strong enhanced anti-cancer effect was achieved by the combination compared with that of each alone, supporting that targeting de novo fatty acid synthesis is an effective approach to potentiate cancer cells to ABT-737. These results supported inhibition of lipogenesis as an additional mechanism contributed to the sensitization effect of GCM in addition to its inhibitory effect on TOPK-survivin axis. However, a recent study by Nelson et al. shows that inhibition of hepatic lipogenesis by liver specific-knockout of ACC leads to an increased liver tumor incidence in comparison with the control in a chemical-induced carcinogenesis model [31], supporting a protective role of ACC-mediated lipogenesis in tumorigenesis. This controversial role of ACC-mediated lipogenesis in carcinogenesis maybe associated with the stages of tumorigenesis. We speculated that lipogenesis prevents normal cell transformation or cancer initiation by regulating oxidative stress but promotes tumor progression in the late stage by fueling the cancer cells. This hypothesis clearly needs to be tested in the future study. 

Bcl-xl is indispensable for platelet survival [32], thus, it is not surprising that BH3 mimetics induce platelet suppression, which poses a major hurdle for their clinical use. In the present study, we demonstrated that the combination resulted in attenuated toxicity on platelets suppression, providing an additional valuable attribute for GCM as a combinatory agent with ABT-737. The detailed mechanisms underlying the protective effect of GCM on platelet is being investigated.

Our previous study has shown that GCM inhibits palmitate-induced lipoapoptosis in a number of liver cells [33]. In the present study, we found that GCM can sensitize liver cancer cells but not non-malignant liver cells to ABT-737-induced apoptosis. These data suggest that GCM can either exert pro-survival or pro-death activity depending on the context. The determinants that govern the decision between the pro-survival or pro-death activity of GCM might be associated with the signaling pathways activated by the stimuli. For examples, palmitate induced lipoapoptosis via inactivating autophagy and inducing ER stress, whereas GCM offered the protection through activating autophagy and inhibiting ER stress. ABT-737 induced cell death by targeting anti-apoptotic Bcl-2 family proteins, whereas GCM potentiated the cancer cells to ABT-737 through suppressing TOPK/survivin axis and inhibiting de novo lipogenesis.

## 5. Conclusions

**Figure 7:**
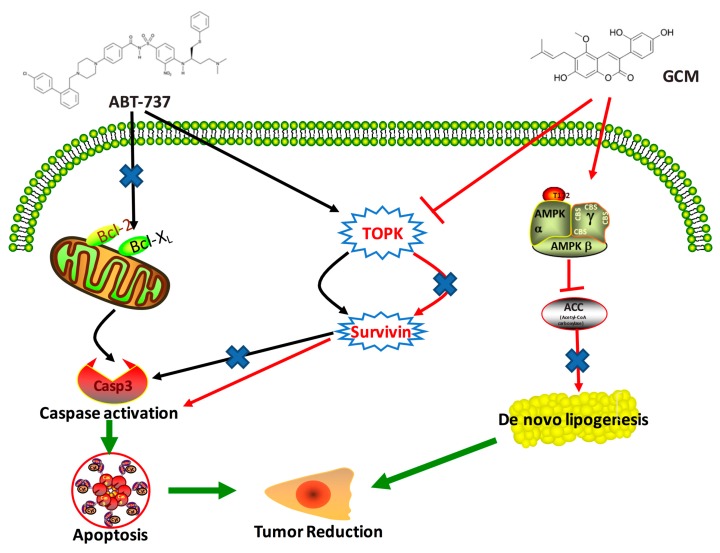
Potential mechanisms involved in the enhanced anti-cancer effect of the combination of GCM and ABT-737. ABT-737 induces apoptosis by targeting anti-apoptotic Bcl-2 family proteins, leading to disruption of the mitochondrial membrane potential, activating caspase, and inducing apoptosis. In the meantime, ABT-737 activates TOPK, resulting in up-regulating survivin, which confers the cancer cells resistant to ABT-737. GCM potentiates the cancer cells to ABT-737 by suppressing TOPK/survivin axis and inhibiting de novo lipogenesis through activating AMPK pathway.

GCM can potentiate liver cancer cells to ABT-737-induced apoptosis in both cell culture and animal models by targeting TOPK-survivin pro-survival signaling pathway and inhibiting ACC-mediated de novo lipogenesis (Figure 7). The findings of the present study implicate that bioactive coumarin compound GCM holds great potential to be used as a novel chemo-potentiating agent for improvement of ABT-737-based therapy.
